# Peripheral neutrophil count and kidney outcomes in IgA nephropathy: a retrospective cohort study of 9,030 patients

**DOI:** 10.3389/fimmu.2026.1952279

**Published:** 2026-10-29

**Authors:** Wei-Bo Le, Jing-Song Shi, Feng Liu, Zhen Cheng

**Affiliations:** National Clinical Research Center of Kidney Diseases, Jinling Hospital, Nanjing University School of Medicine, Nanjing, China

**Keywords:** estimated glomerular filtration rate, IgA nephropathy, kidney outcomes, neutrophil, proteinuria

## Abstract

**Background:**

Peripheral neutrophil (NEU) count is routinely measured in patients with immunoglobulin A (IgA) nephropathy (IgAN). However, its association with kidney outcomes remains unclear.

**Methods:**

This retrospective, single-center study included 9,030 adults with biopsy-proven primary IgAN. Sequential Cox models compared NEU ≥4.0 *versus* < 4.0 × 10^9^/L using the rounded cohort median as the descriptive cutoff. Basic models were adjusted for age, sex, hypertension, and diabetes. Proteinuria and estimated glomerular filtration rate (eGFR) were then added separately and jointly. Sex, an antecedent factor, was examined as an illustrative comparison. Exploratory *g*-computation was used to estimate the proteinuria-related and remaining components of between-group differences in the 10-year outcome probabilities. Cross-sectional multinomial models examined report-derived crescent categories.

**Results:**

Over a median follow-up of 9.3 years to kidney failure or censoring, 1,368 patients developed kidney failure, and 2,186 experienced a ≥50% decline in eGFR. For these outcomes, the basic-adjusted hazard ratios (HRs) comparing higher *versus* lower NEU were 1.22 (95%CI = 1.09–1.35) and 1.24 (95%CI = 1.14–1.35), respectively. These HRs attenuated to 1.00 (95%CI = 0.90–1.11) and 1.04 (95%CI = 0.95–1.13) after adjustment for proteinuria. The association with male sex also weakened or reversed after adjustment for kidney disease markers. The proteinuria-related components for NEU were 2.680 (95%CI = 2.221–3.125) and 3.623 (95%CI = 3.038–4.199) percentage points, with the remaining component intervals including zero for both outcomes. Sex also showed positive proteinuria-related components but retained a positive remaining component for ≥50% decline in eGFR. Among 8,968 patients with classifiable crescent lesions, higher NEU was associated with C1 and C2 categories *versus* C0, including after adjustment for proteinuria and eGFR.

**Conclusion:**

A higher NEU count is associated with adverse kidney outcomes, with marked attenuation after adjustment for proteinuria. This attenuation is consistent with a substantial overlap between the NEU count and the markers of disease severity, particularly proteinuria. Whether proteinuria lies on a causal pathway cannot be determined from these data.

## 1 Introduction

Immunoglobulin A (IgA) nephropathy (IgAN) is the most common primary glomerular disease worldwide, and approximately 20%–40% of patients develop kidney failure within 20 years of diagnosis (1–3). Its pathogenesis is commonly described by the “four-hit” model: increased production of galactose-deficient IgA1, formation of anti-glycan autoantibodies, formation and mesangial deposition of immune complexes, and subsequent complement-mediated inflammation and kidney injury (3, 4). Although this framework has clarified the sequence of IgAN-specific immune events, the contribution of innate immune cells, particularly neutrophils (NEU), to disease severity and progression remains incompletely understood.

Genome-wide association studies across Asian and European populations have identified approximately 30 IgAN susceptibility loci and multiple immune pathways extending beyond IgA production and complement regulation (5–7). Cell-type enrichment analyses have highlighted peripheral blood NEU, and non-HLA signals include those at the *FCAR*, *ITGAM*, *CARD9*, *PADI4*, and the *DEFA* regions (6–8). Fine-mapping and follow-up studies of the *DEFA* locus have reported independent variants and copy number variant associations, although the functional consequences remain unresolved (9–11). The functions of the genes at these loci are consistent with IgA immune complex recognition through FcαRI/CD89, β2 integrin-dependent adhesion and trafficking, inflammatory signaling, neutrophil extracellular trap (NET) formation, and antimicrobial responses (12, 13). Although these associations do not establish effector cell specificity or a causal cascade, they support investigating NEU in IgAN.

Peripheral blood cell counts provide readily available measures of systemic inflammation. In IgAN, higher neutrophil-to-lymphocyte ratios (NLRs) have been associated with more severe clinical and pathological features and a greater risk of kidney disease progression (14, 15). However, a ratio cannot distinguish the prognostic contribution of its individual components. Therefore, this study focused on absolute NEU count. This distinction matters because NEU have distinct effector functions in antibody-mediated inflammation, including the release of NETs (16). Experimental studies have shown that IgA immune complexes can induce FcαRI-dependent NET formation (17–19). In IgAN, circulating markers of NET formation, including citrullinated histone H3, have been reported to be elevated and to correlate with galactose-deficient IgA1. Experimental inhibition of NET formation has also reduced kidney injury (20). These observations provide a biological rationale for examining the absolute NEU count in relation to kidney outcomes in IgAN.

Interpreting the association between NEU count and kidney outcomes requires consideration of proteinuria and kidney function. Proteinuria is one of the strongest predictors of kidney failure in IgAN and is included in validated prognostic models (21, 22). A reduction in proteinuria has also been supported as a reasonably likely surrogate endpoint in clinical trials (23). When evaluating factors that may act upstream of kidney injury, adjustment for proteinuria and estimated glomerular filtration rate (eGFR) must be interpreted with caution. Assessing the role of these factors in disease progression differs from determining whether they improve prediction beyond the established clinical markers (24).

We therefore evaluated the association of the baseline NEU count with kidney failure and a ≥50% decline in eGFR in adults with biopsy-proven primary IgAN and examined how these associations changed after adjustment for proteinuria and eGFR, separately and jointly. In the exploratory analyses, we used sex, an antecedent factor, as an illustrative comparison to examine how adjustment for kidney disease markers can alter associations with such a factor. Finally, we used exploratory *g*-computation to estimate the proteinuria-related and remaining components of between-group differences in the 10-year outcome probabilities for both NEU count and sex. We also examined the cross-sectional association between NEU and crescent categories derived from baseline biopsy reports.

## 2 Materials and methods

### 2.1 Study design and population

This retrospective, single-center cohort study was conducted at the National Clinical Research Center of Kidney Diseases, Jinling Hospital, Nanjing University School of Medicine. We screened patients with biopsy-proven primary IgAN diagnosed between August 2007 and August 2016. Eligible patients were ≥18 years of age at biopsy, had a baseline eGFR of at least 30 ml/min per 1.73 m^2^, and either had at least 1 year of adjudicated follow-up or developed kidney failure within the first year. The exclusion criteria were minimal change disease, podocytopathy, a coexisting independent pathological diagnosis of focal segmental glomerulosclerosis (FSGS), another coexisting or secondary glomerular disease, corticosteroid or immunosuppressive therapy before biopsy, or missing baseline NEU count or 24-h urinary protein excretion (24hUP, in grams per day).

The study was conducted in accordance with the Declaration of Helsinki and was approved by the Institutional Review Board of Jinling Hospital, Nanjing University School of Medicine (no. 2026DZKY-081-01). The requirement for informed consent was waived because this retrospective study used anonymized medical records and posed no more than minimal risk to participants’ rights and welfare.

### 2.2 Data collection and definitions

Demographic, clinical, and laboratory data were collected relative to the kidney biopsy date. Hematologic variables were obtained from complete blood counts and included white blood cell (WBC), NEU, lymphocyte, and platelet counts. eGFR was calculated using the 2021 race-free CKD-EPI creatinine equation (25). Hypertension was defined as a systolic blood pressure of at least 140 mmHg, a diastolic blood pressure of at least 90 mmHg, or use of antihypertensive medication. Diabetes was defined as a history of diabetes or an admission glycated hemoglobin (HbA1c) ≥6.5%. Post-biopsy therapies were not included in the baseline model sequence because their initiation and modification occurred after exposure assessment and could depend on evolving disease severity; conventional baseline adjustment would not address this time-dependent treatment process. Baseline light microscopy reports were used to extract the proportion of glomeruli with cellular or fibrocellular crescents and classify involvement as C0 (none), C1 (>0% to <25%), or C2 (≥25%).

### 2.3 Outcomes

The primary outcome was kidney failure, defined as an eGFR <15 ml/min per 1.73 m^2^, initiation of dialysis, kidney transplantation, or death due to kidney failure. The secondary outcome was a ≥50% decline in eGFR from baseline. Follow-up data were updated through August 2025, and outcomes were reviewed on a case-by-case basis by specialist physicians using laboratory reports and electronic medical records. For time-to-event analyses, the biopsy date served as time zero, and patients without the outcome were censored at their last adjudicated outcome-specific follow-up, including verified clinical follow-up without a contemporaneous laboratory measurement. Data on non-kidney death were insufficient for competing-risk analysis.

### 2.4 Statistical analysis

Continuous variables are summarized as the mean ± standard deviation or median (interquartile range), while categorical variables are presented as counts (%). Groups were compared using Student’s *t*-test, the Wilcoxon rank-sum test, or Pearson’s chi-squared test. The NEU distributions across C0–C2 were compared using the Kruskal–Wallis test. Spearman’s correlations were used to assess the associations of NEU with log-proteinuria and eGFR. Patients with missing core covariates were excluded before cohort definition. Analyses of body mass index (BMI), smoking, and C-reactive protein (CRP) used available-data subsets without imputation.

Kidney survival was assessed using Kaplan–Meier curves and log-rank tests. Cox models estimated the hazard ratios (HRs) and 95% confidence intervals (CIs) for NEU ≥4.0 *versus* < 4.0 × 10^9^/L using the rounded cohort median as the cutoff. Exploratory multinomial logistic models estimated the odds ratios (ORs) and the 95% CIs for C1 and C2 *versus* C0 using the same binary NEU contrast and, separately, per 1 × 10^9^/L increase. Unadjusted and basic-adjusted restricted cubic splines described continuous NEU associations with kidney outcomes, with knots at the 5th, 35th, 65th, and 95th percentiles and the median as the reference. Likelihood ratio tests assessed the overall and nonlinear spline associations.

Cox regression analyses of NEU and kidney outcomes and multinomial logistic regression analyses of NEU and crescent categories used five models: model 1, unadjusted; model 2, adjusted for age, sex, hypertension, and diabetes; model 3, model 2 plus proteinuria; model 4, model 2 plus eGFR; and model 5, model 2 plus both proteinuria and eGFR. All five models used the same participants within each analysis (9,030 for the kidney outcome analyses and 8,968 for the crescent analyses). For each kidney outcome, the event and censoring times were identical across the five Cox models. Age and eGFR were entered linearly, and 24hUP was natural log-transformed. This sequence examined associations conditional on kidney disease markers (24, 26). The observed attenuation prompted an illustrative male-*versus*-female comparison using the same sequence, with basic adjustment for age, hypertension, and diabetes, excluding NEU. Covariate-specific and global scaled Schoenfeld tests assessed proportional hazards.

After the observed attenuation, we used exploratory *g*-computation to estimate the proteinuria-related and remaining components of the 10-year outcome probability differences for the same NEU and sex contrasts. A linear log-proteinuria model was paired with an annual piecewise exponential outcome model with log-scale baseline hazards. The outcome model included binary exposure, log-proteinuria, their interaction, and terms that allowed the associations of exposure and proteinuria with the outcome to vary over follow-up. Both models were adjusted for age, sex, hypertension, and diabetes in the NEU analysis and for age, hypertension, and diabetes in the sex analysis. These probability components did not decompose HR attenuation.

Using centered empirical residuals to generate predicted proteinuria distributions, we first estimated the overall difference in the standardized 10-year outcome probabilities between the high- and low-NEU groups, with each group assigned its own predicted proteinuria distribution. We then estimated the remaining difference with both groups assigned the predicted proteinuria distribution of the low-NEU group. Subtracting the remaining difference from the overall difference yielded the proteinuria-related component. Components were expressed in percentage points. The same procedure was applied to sex, with women as the reference group.

Percentile 95% CIs used 2,000 patient bootstrap resamples, refitting both models. Failed attempts were not replaced. These exploratory analyses and the 10-year framework were developed during revision. Causal interpretation requires consistency, positivity, correct model specification, conditionally non-informative censoring, no unmeasured confounding of the exposure–outcome, exposure–proteinuria, or proteinuria–outcome relationships, and no exposure-induced confounder of the proteinuria–outcome relationship (27). Temporal ordering was not established, and CIs captured sampling variations rather than confounding or model selection uncertainty.

Sensitivity analyses added BMI (linear or spline), smoking (current/former versus never), or CRP (≤3, >3 to ≤10, or >10 mg/L), comparing models on identical available-data subsets with and without proteinuria adjustment. The CRP categories used upper bounds, with lower-bound assignments as a sensitivity check. Continuous log(1 + CRP) models used bounded summaries, assigning measurements below a reporting limit to zero, half the limit, or the limit. Cox sensitivity models allowed covariate coefficients to vary across 0–5, >5–10, and >10 years, and likelihood-ratio tests assessed additional period-specific NEU effects. Basic-adjusted crescent sensitivity analyses restricted the biopsies to at least eight glomeruli or included fibrous crescents, with the two definitions also compared in the same patients.

Continuous-NEU probability component sensitivity models used the same four-knot splines, spline–proteinuria interactions, and time-varying terms, retaining the cohort, adjustment set, 10-year horizon, and bootstrap procedure. The contrast was Q75 *versus* Q25 (5.02 *vs*. 3.22 × 10^9^/L).

Analyses used R 4.5.3 with the survival, splines, and nnet packages. The HR and OR contrasts and their CIs used the Wald method, while the overall crescent associations used two-degree-of-freedom likelihood-ratio tests. All tests were two-sided, with nominal *p*-values and no multiplicity adjustment. Incremental predictive performance was not evaluated.

## 3 Results

### 3.1 Study population and baseline characteristics

Cohort selection is summarized in **Figure 1**. Among 13,925 patients with biopsy-confirmed IgAN diagnosed between August 2007 and August 2016, 12,901 met the adult age criterion and 12,136 had a baseline eGFR ≥30 ml/min per 1.73 m^2^. Of these, 10,371 had at least 1 year of adjudicated follow-up or developed kidney failure within the first year. Patients were then excluded for minimal change disease, podocytopathy, a coexisting independent pathological diagnosis of FSGS (*n* = 239), or for coexisting or secondary glomerular disease (*n* = 158). Further exclusions comprised corticosteroid or immunosuppressive treatment before biopsy (*n* = 713), missing baseline NEU count or 24hUP (*n* = 211), and missing other core covariates (hypertension or diabetes; *n* = 20). The final cohort included 9,030 patients, whose baseline characteristics are presented in **Table 1**.

**Figure 1:**
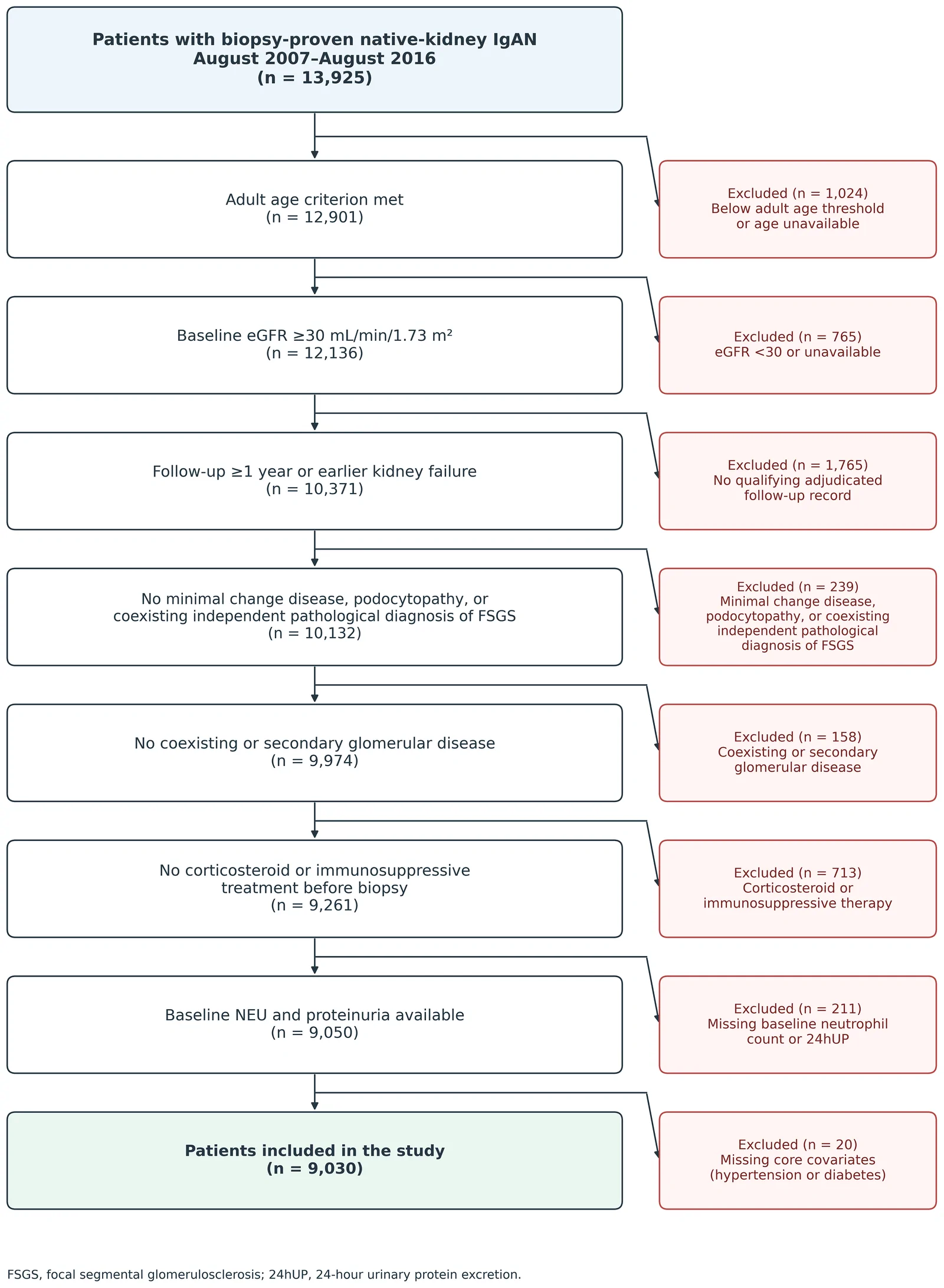
Cohort assembly. Patients with biopsy-proven native-kidney immunoglobulin A nephropathy (IgAN) were screened sequentially for adult age, baseline eGFR ≥30 ml/min per 1.73 m^2^, and at least 1 year of adjudicated follow-up or kidney failure within the first year. Subsequent exclusions addressed competing pathology, documented pre-biopsy corticosteroid or immunosuppressive treatment, missing baseline neutrophil (NEU) or proteinuria (*n* = 211), and missing core covariates (hypertension or diabetes; *n* = 20). The final study cohort comprised 9,030 patients. BMI and smoking availability did not determine inclusion. Follow-up eligibility uses adjudicated kidney failure or censoring time, not the last laboratory date. Missing adjudicated follow-up is distinguished from a known duration below 1 year.

**Table 1:** Baseline characteristics of 9,030 patients with IgA nephropathy, overall and stratified by baseline neutrophil count (<4.0 *vs*. ≥4.0 × 10^9^/L).

Variable	Overall: *N* = 9,030	NEU <4.0: *n* = 4,446	NEU ≥4.0: *n* = 4,584	*p*-value
Male sex, *n* (%)	4,278 (47.4)	1,948 (43.8)	2,330 (50.8)	<0.001
Age at biopsy (years)	35.8 ± 10.4	36.0 ± 10.5	35.6 ± 10.3	0.074
Hypertension, *n* (%)	3,812 (42.2)	1,694 (38.1)	2,118 (46.2)	<0.001
Current or former smoker, *n* (%)	1,351 (15.2)	574 (13.1)	777 (17.2)	<0.001
Diabetes, *n* (%)	378 (4.2)	168 (3.8)	210 (4.6)	0.057
BMI (available records) (kg/m^2^)	23.0 ± 3.5	22.7 ± 3.3	23.4 ± 3.6	<0.001
WBC (×10^9^/L)	6.92 ± 1.90	5.61 ± 0.95	8.19 ± 1.71	<0.001
Neutrophils (×10^9^/L)	4.30 ± 1.61	3.12 ± 0.60	5.44 ± 1.47	<0.001
Neutrophils (%)	61.0 ± 8.9	55.9 ± 7.3	66.1 ± 7.2	<0.001
Lymphocytes (×10^9^/L)	2.07 ± 0.60	1.98 ± 0.55	2.15 ± 0.64	<0.001
Lymphocytes (%)	30.8 ± 7.9	35.2 ± 6.8	26.5 ± 6.4	<0.001
Same-report NLR	2.04 ± 1.09	1.66 ± 0.77	2.41 ± 1.23	<0.001
Platelets (×10^9^/L)	220.2 ± 62.9	207.0 ± 58.8	233.1 ± 64.1	<0.001
eGFR (ml/min per 1.73 m^2^)	91.6 ± 28.0	94.1 ± 27.6	89.3 ± 28.2	<0.001
24hUP (g/day)	0.86 (0.53–1.42)	0.77 (0.47–1.23)	0.99 (0.60–1.61)	<0.001
Serum albumin (g/L)	41.8 ± 4.7	41.8 ± 4.5	41.7 ± 4.9	0.396
Uric acid (μmol/L)	373.4 ± 100.4	360.9 ± 97.2	385.5 ± 101.9	<0.001
Serum C3 (g/L)	1.00 ± 0.21	0.95 ± 0.19	1.04 ± 0.22	<0.001
Serum C4 (g/L)	0.21 ± 0.07	0.20 ± 0.07	0.22 ± 0.08	<0.001
Serum IgA (g/L)	2.94 ± 1.00	2.92 ± 0.99	2.96 ± 1.02	0.065
Serum IgG (g/L)	11.11 ± 2.77	11.36 ± 2.68	10.86 ± 2.83	<0.001
Time to kidney failure or censoring (years)	9.3 (5.1–12.3)	9.4 (5.1–12.4)	9.3 (5.1–12.2)	0.433
Progression to kidney failure, *n* (%)	1,368 (15.1)	582 (13.1)	786 (17.1)	<0.001
50% decline in eGFR, *n* (%)	2,186 (24.2)	934 (21.0)	1,252 (27.3)	<0.001

Values are the mean ± SD, median (IQR), or *n* (%). Percentages are based on non-missing denominators. The *p*-values compare the NEU groups using Student’s *t*-test for the mean ± SD, the Wilcoxon rank-sum test for the median (IQR) and Pearson’s chi-squared test for *n* (%) based on available observations. Outcome count *p*-values compare crude proportions, not time-to-event associations. Missing counts are provided in the accompanying data file. The cutoff of 4.0 × 10^9^/L was based on the rounded cohort median and used to define the descriptive NEU groups. NLR was calculated using neutrophils and lymphocytes from the same report.

24hUP, 24-h urinary protein excretion; BMI, body mass index; eGFR, estimated glomerular filtration rate; NEU, neutrophil count; NLR, neutrophil-to-lymphocyte ratio; WBC, white blood cell count.

At biopsy, the mean age was 35.8 ± 10.4 years, and 4,278 (47.4%) participants were men. The mean eGFR was 91.6 ± 28.0 ml/min per 1.73 m^2^, and the median 24hUP was 0.86 g/day (IQR = 0.53–1.42). The mean WBC, NEU, and lymphocyte counts were 6.92 ± 1.90, 4.30 ± 1.61, and 2.07 ± 0.60 × 10^9^/L, respectively. For descriptive comparisons using the rounded median baseline NEU count (4.0 × 10^9^/L) as the cutoff, 4,584 patients had higher and 4,446 had lower NEU counts (**Table 1**). Compared with those in the lower-NEU group, patients with higher counts were more often men (50.8% *vs*. 43.8%) and hypertensive (46.2% *vs*. 38.1%), had lower eGFR (89.3 ± 28.2 *vs*. 94.1 ± 27.6 ml/min per 1.73 m^2^), and had higher 24hUP [median = 0.99 (0.60–1.61) *vs*. 0.77 (0.47–1.23) g/day]. They also exhibited higher serum uric acid, C3, and C4 and lower immunoglobulin G (IgG). Neutrophil count was positively correlated with log-transformed proteinuria (Spearman’s *ρ* = 0.212) and inversely correlated with eGFR (*ρ* = −0.111).

The median follow-up to kidney failure or censoring was 9.3 years (IQR = 5.1–12.3), with 1,368 (15.1%) patients developing kidney failure and 2,186 (24.2%) reaching the ≥50% eGFR decline endpoint. This median follow-up time was similar between groups (9.3 *vs*. 9.4 years). The proportions of patients who developed kidney failure (17.1% *vs*. 13.1%) and had ≥50% eGFR decline (27.3% *vs*. 21.0%) were higher in those with NEU counts ≥4.0 × 10^9^/L.

### 3.2 Associations between neutrophil count and kidney outcomes

Patients with NEU counts ≥4.0 × 10^9^/L had lower Kaplan–Meier event-free survival for both kidney failure and ≥50% eGFR decline (log-rank *p* < 0.001 for both outcomes) (**Figures 2A, B**). Unadjusted Cox models comparing these groups yielded HRs of 1.33 (95%CI = 1.20–1.48) for kidney failure and 1.34 (95%CI = 1.23–1.45) for ≥50% eGFR decline (both *p* < 0.001) (**Figure 3A**). The unadjusted spline curves showed departures from linearity for both kidney failure (*p*-value for nonlinearity = 0.03) (**Figure 2C**) and ≥50% eGFR decline (*p*-value for nonlinearity < 0.001) (**Figure 2D**). For the latter outcome, the HRs increased up to approximately 6.0 × 10^9^/L and rose more gradually thereafter.

**Figure 2:**
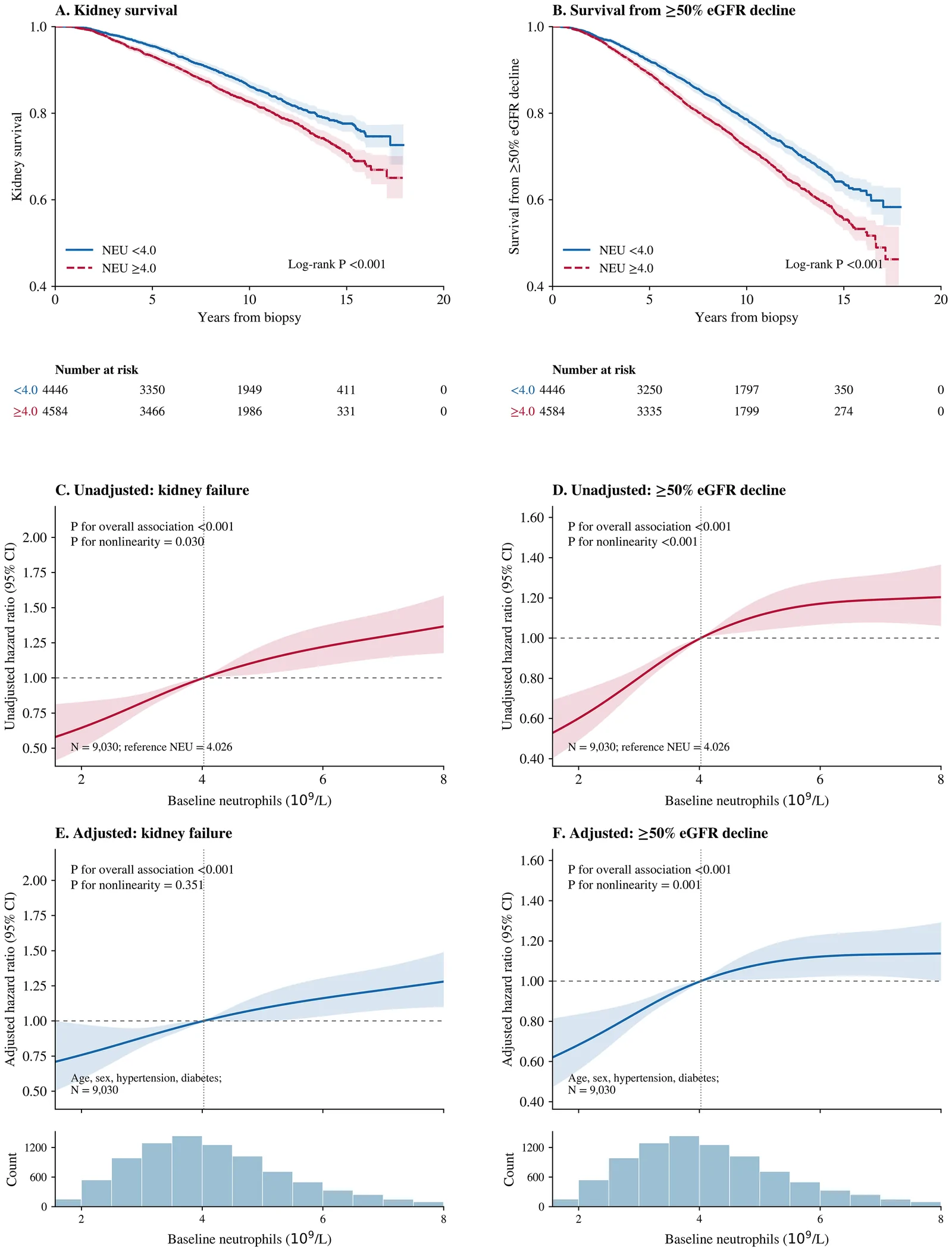
Kaplan–Meier curves and unadjusted and adjusted spline associations of the baseline neutrophil count with kidney outcomes. **(A, B)** Kidney survival **(A)** and survival free from ≥50% estimated glomerular filtration rate (eGFR) decline **(B)** in 9,030 patients. Groups are neutrophil (NEU) <4.0 × 10^9^/L (*n* = 4,446) and ≥4.0 × 10^9^/L (*n* = 4,584). Follow-up starts at biopsy and uses endpoint-specific verified event and censoring times. Shaded bands indicate 95% confidence intervals. **(C, D)** Unadjusted restricted cubic spline associations with kidney failure **(C)** and ≥50% eGFR decline **(D)**. **(E, F)** Corresponding associations adjusted for age, sex, hypertension, and diabetes. All spline models use the same 9,030 participants and four fixed knots at the 5th, 35th, 65th, and 95th percentiles of NEU. Shaded bands are pointwise 95% confidence intervals, with the reference NEU being 4.0 × 10^9^/L. Overall association *p*-values jointly test all NEU spline terms, while nonlinearity *p*-values test the nonlinear spline terms. The histograms below **(E, F)** show the NEU distribution in the 9,030 study participants, shared by **(C–F)**, respectively. Hazard ratios (HRs) comparing NEU ≥4.0 versus < 4.0 × 10^9^/L across adjustment models are summarized in **Figure 3A**.

**Figure 3:**
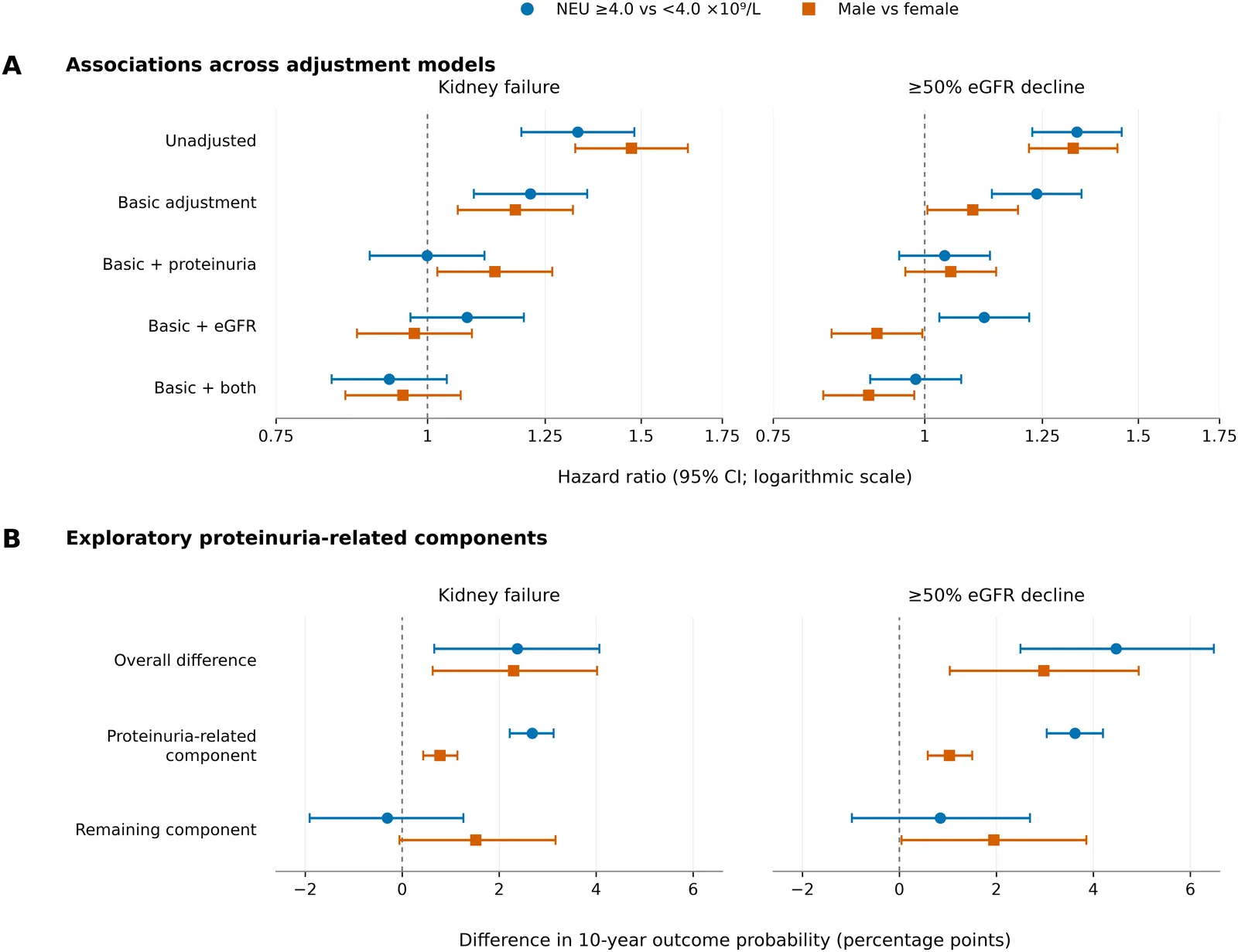
Changes in neutrophil (NEU) and sex associations across adjustment models and exploratory proteinuria-related components. Points and lines show estimates and 95% CIs in 9,030 participants: blue circles, NEU ≥4.0 *versus* < 4.0 × 10^9^/L; orange squares, men versus women. Left, kidney failure; right, ≥50% eGFR decline. **(A)** Hazard ratios (HRs) across five adjustment models (logarithmic axis; reference line, 1). Basic adjustment includes age, hypertension, and diabetes, plus sex for NEU models; sex models exclude NEU. **(B)** Model-estimated 10-year probability differences in percentage points (reference line, 0). The overall difference compares groups with their respective modeled proteinuria distributions. The remaining component assigns both groups the reference group distribution (low NEU or female sex); the proteinuria-related component is the difference between these contrasts. The overall difference equals the proteinuria-related component plus the remaining component; confidence limits are not additive. Models use the basic adjustment sets in **(A)**, without the estimated glomerular filtration rate (eGFR). CIs use 2,000 patient bootstrap resamples. The 10-year horizon does not require 10 years of follow-up for every participant. Sex is included as an illustrative antecedent factor comparison. Panel **(B)** does not decompose the HR attenuation in **(A)** or establish mediation. Model assumptions are described in “*Materials and methods*.”.

These associations persisted after adjustment for age, sex, hypertension, and diabetes. In the spline models, the overall association *p*-values were <0.001 for both outcomes, whereas the *p*-values for nonlinearity were 0.3505 and 0.0015, respectively (**Figures 2E, F**). Despite departures from linearity in some models, the unadjusted and basic-adjusted curves increased monotonically throughout the displayed NEU range up to 8.0 × 10^9^/L (**Figures 2C–F**), indicating changes in slope rather than a reversal in the direction of association. The CIs widened at sparse NEU counts.

### 3.3 Changes in neutrophil and sex associations across adjustment models

We next examined how the addition of proteinuria or eGFR to the basic models changed these associations. For NEU ≥4.0 *versus* < 4.0 × 10^9^/L, the basic-adjusted HRs were 1.22 (95%CI = 1.09–1.35, *p* < 0.001) for kidney failure and 1.24 (95%CI = 1.14–1.35, *p* < 0.001) for ≥50% eGFR decline. The addition of proteinuria attenuated the HRs to 1.00 (95%CI = 0.90–1.11, *p* = 0.989) and 1.04 (95%CI = 0.95–1.13, *p* = 0.392), respectively. The addition of eGFR alone yielded HRs of 1.08 (95%CI = 0.97–1.20) and 1.12 (95%CI = 1.03–1.22), while joint adjustment yielded 0.93 (95%CI = 0.83–1.04) and 0.98 (95%CI = 0.90–1.07), respectively (**Figure 3A**).

The attenuation observed for NEU prompted us to examine whether adjustment for kidney disease markers also altered the associations with sex, an antecedent factor. The unadjusted male-*versus*-female HRs were 1.47 (95%CI = 1.32–1.64, *p* < 0.001) for kidney failure and 1.33 (95%CI = 1.22–1.44, *p* < 0.001) for ≥50% eGFR decline. After adjustment for age, hypertension, and diabetes, the corresponding HRs were 1.18 (95%CI = 1.06–1.32, *p* = 0.003) and 1.10 (95%CI = 1.00–1.19, *p* = 0.038). The addition of proteinuria yielded HRs of 1.14 (95%CI = 1.02–1.27, *p* = 0.022) and 1.05 (95%CI = 0.96–1.15, *p* = 0.261), while addition of eGFR instead yielded 0.98 (95%CI = 0.87–1.09, *p* = 0.655) and 0.91 (95%CI = 0.84–1.00, *p* = 0.039). With both proteinuria and eGFR included, the HRs were 0.95 (95%CI = 0.86–1.07, *p* = 0.405) and 0.90 (95%CI = 0.82–0.98, *p* = 0.016), respectively. All models used the same participants and endpoint-specific follow-up.

Thus, both NEU and male sex were associated with higher risks in the basic models; however, these associations weakened or shifted to below an HR of 1 after adjustment for kidney disease markers. The pattern differed by marker: the NEU associations attenuated more after the addition of proteinuria, whereas the sex associations changed more after the addition of eGFR (**Figure 3A**).

### 3.4 Exploratory analysis of proteinuria-related probability components

The greater attenuation of the NEU association after proteinuria adjustment prompted us to explore a proteinuria-related explanation. We therefore estimated the proteinuria-related and remaining components of the modeled 10-year outcome probability differences, applying the same framework to NEU and sex (**Figure 3B**). For NEU ≥4.0 *versus* < 4.0 × 10^9^/L, the overall differences were 2.375 (95%CI = 0.661–4.067) percentage points for kidney failure and 4.469 (95%CI = 2.497–6.485) for ≥50% eGFR decline. The corresponding proteinuria-related components were 2.680 (95%CI = 2.221–3.125) and 3.623 (95%CI = 3.038–4.199) percentage points. The remaining components were −0.305 (95%CI = −1.908 to 1.264) and 0.846 (95%CI = −0.981 to 2.692) percentage points, respectively. For example, the kidney failure overall difference of 2.375 percentage points is the sum of a positive proteinuria-related component of 2.680 and a remaining component of −0.305. For both outcomes, the remaining component CIs included zero, indicating no clear remaining difference under these modeled scenarios.

For male *versus* female sex, the overall differences were 2.298 (95%CI = 0.628–4.020) percentage points for kidney failure and 2.981 (95%CI = 1.042–4.933) for ≥50% eGFR decline. The corresponding proteinuria-related components were 0.780 (95%CI = 0.434–1.140) and 1.031 (95%CI = 0.584–1.501) percentage points, while those for the remaining components were 1.518 (95%CI = −0.056–3.162) and 1.950 (95%CI = 0.044–3.855) percentage points, respectively. Both exposures therefore had positive proteinuria-related components, but with the remaining component for sex also positive for ≥50% eGFR decline, with a CI excluding zero. All 2,000 patient bootstrap resamples for each exposure and outcome passed the fitting and prediction checks. These exploratory components do not establish biological temporal ordering or proteinuria as a causal mediator.

### 3.5 Sensitivity analyses

Covariate sensitivity analyses used the NEU ≥4.0 *versus* < 4.0 × 10^9^/L contrast. The addition of BMI, smoking, or CRP separately had little effect on the basic-adjusted associations within the same available-data subsets (8,875, 8,903, and 8,009 participants, respectively). With BMI and proteinuria included, the HRs were 1.01 (95%CI = 0.91–1.13) for kidney failure and 1.05 (95%CI = 0.96–1.15) for ≥50% eGFR decline. With smoking and proteinuria included, the HRs were 0.99 (95%CI = 0.89–1.11) for kidney failure and 1.04 (95%CI = 0.95–1.13) for ≥50% eGFR decline. With CRP and proteinuria included, the HRs were 1.03 (95%CI = 0.92–1.15) for kidney failure and 1.05 (95%CI = 0.96–1.15) for ≥50% eGFR decline. Thus, attenuation after proteinuria adjustment remained evident after accounting for each additional factor.

Global Schoenfeld tests indicated departures from proportional hazards, whereas the NEU term showed no clear departure, prompting sensitivity analyses that allowed adjustment-variable coefficients to vary over time. Allowing adjustment-variable coefficients to vary across 0–5, >5–10, and >10 years yielded proteinuria-adjusted binary NEU HRs of 1.00 (95%CI = 0.90–1.12) for kidney failure and 1.04 (95%CI = 0.95–1.13) for ≥50% eGFR decline. Further allowing NEU effects to vary across these periods did not clearly improve model fit (likelihood ratio *p* = 0.491 and *p* = 0.730, respectively).

The continuous-spline sensitivity analysis showed the same qualitative pattern as the binary analysis: the proteinuria-related components were positive for both outcomes, whereas the remaining component CIs included zero. These findings indicate that the pattern was not confined to the 4.0 × 10^9^/L grouping.

### 3.6 Peripheral neutrophil count and crescent severity

Crescent categories could be assigned to 8,968 patients: 5,496 were C0, 3,315 were C1, and 157 were C2. There were 62 patients with an unknown category who were excluded from this analysis. The median NEU counts were 3.94 (IQR = 3.15–4.90), 4.14 (3.32–5.18), and 4.84 (3.93–6.26) × 10^9^/L, respectively. The NEU distributions differed across groups (Kruskal–Wallis *p* < 0.001) (**Figure 4A**). For NEU ≥4.0 *versus* < 4.0 × 10^9^/L, the basic-adjusted ORs were 1.34 (95%CI = 1.22–1.46) for C1 *versus* C0 and 2.96 (95%CI = 2.07–4.23) for C2 *versus* C0 (both *p* < 0.001). After additional adjustment for proteinuria and eGFR, the corresponding ORs were 1.17 (95%CI = 1.07–1.28, *p* < 0.001) and 1.85 (95%CI = 1.27–2.67, *p* = 0.001) (**Figure 4B**).

**Figure 4:**
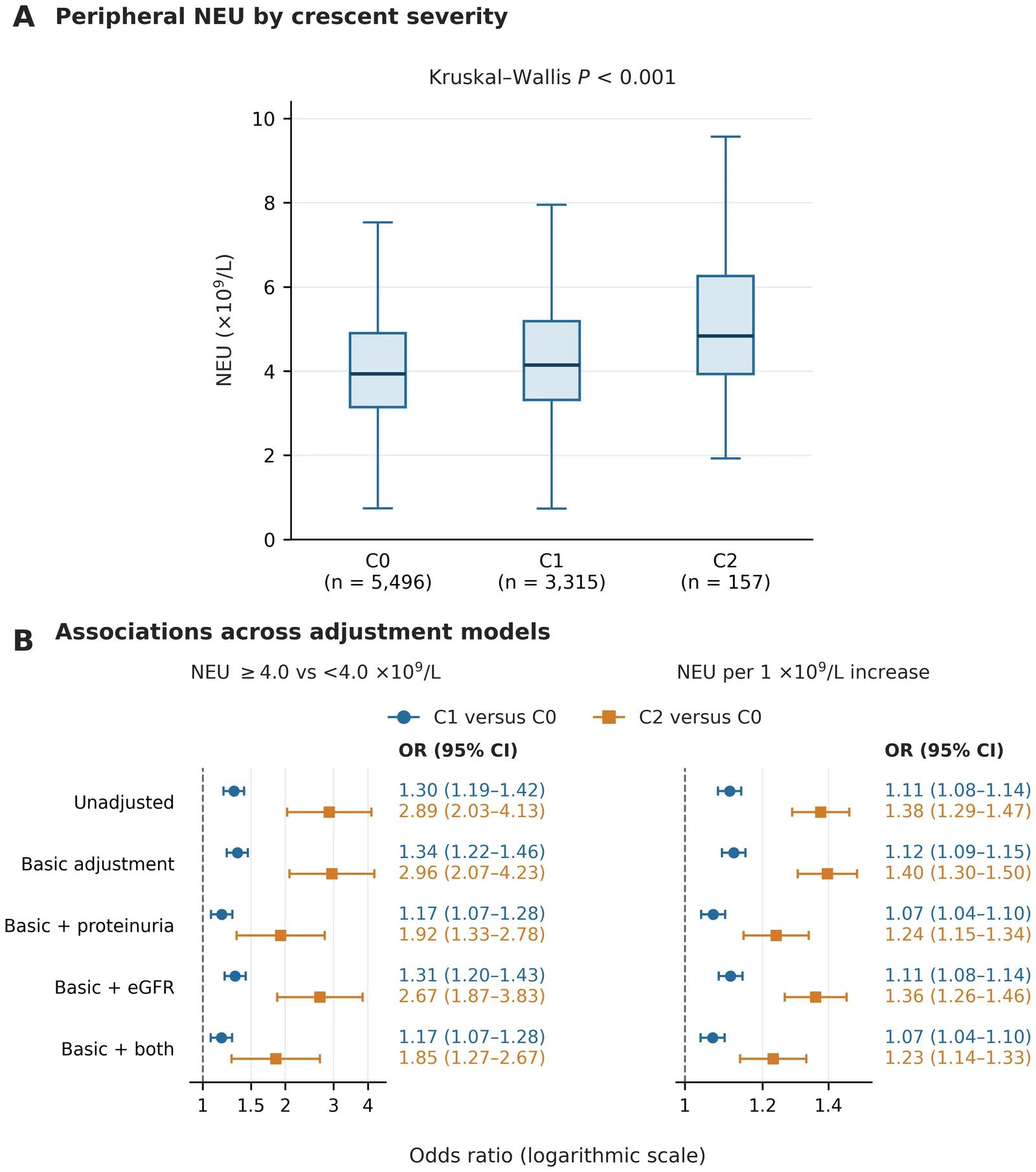
Peripheral neutrophil count and crescent severity in IgA nephropathy. This analysis included 8,968 patients with report-derived crescent categories (C0, *n* = 5,496; C1, *n* = 3,315; C2, *n* = 157), with 62 patients with an unknown crescent category excluded. *C0* denotes the absence of cellular or fibrocellular crescents; *C1* and *C2* denote involvement of >0% to <25% and ≥25% of glomeruli, respectively. **(A)** Box plots of the neutrophil (NEU) count. The *p*-value in **(A)** was obtained from a Kruskal–Wallis test comparing the NEU distributions across the three crescent groups. **(B)** Odds ratios (ORs) for NEU ≥4.0 versus < 4.0 × 10^9^/L (*left*) and per 1 × 10^9^/L increase in NEU count (*right*). In **(B)**, blue circles represent C1 versus C0, and orange squares represent C2 versus C0. Horizontal lines indicate 95% confidence intervals (CIs). The OR axes are logarithmic, with reference lines at 1 and different ranges for the two exposure specifications. All five models included the same 8,968 patients. Overall tests for NEU used two-degree-of-freedom likelihood-ratio tests. Category-specific tests and CIs used the Wald method. *P*-values are exploratory and unadjusted for multiplicity. eGFR, estimated glomerular filtration rate.

Continuous NEU analyses showed the same direction of association (**Figure 4B**). With proteinuria and eGFR included, each 1 × 10^9^/L higher NEU count was associated with ORs of 1.07 (95%CI = 1.04–1.10) for C1 *versus* C0 and 1.23 (95%CI = 1.14–1.33) for C2 *versus* C0 (both *p* < 0.001). The overall NEU association tests were *p* < 0.001 in all five models for both exposure specifications. The direction of association was unchanged in the basic-adjusted sensitivity analyses restricted to biopsies with at least eight glomeruli (*n* = 8,809), using the definition that included fibrous crescents (*n* = 8,900), and comparing both definitions in the same 8,897 patients.

## 4 Discussion

Among 9,030 adults with biopsy-proven IgAN followed for a median of 9.3 years to kidney failure or censoring, NEU ≥4.0 *versus* < 4.0 × 10^9^/L was associated with both kidney failure and ≥50% eGFR decline after adjustment for age, sex, hypertension, and diabetes. Both associations attenuated markedly after the addition of proteinuria. Exploratory *g*-computation yielded positive proteinuria-related components of the model-estimated 10-year outcome probability differences, with no clear remaining difference detected for either outcome. The findings are compatible with, but do not establish, the hypothesized relationship among NEU-related inflammation, proteinuria, and kidney outcomes. Our results extend prior IgAN studies associating higher NLR with greater proteinuria, lower kidney function, more severe pathological lesions, and poorer kidney outcomes (14, 15). Because a ratio combines two cell populations, it cannot attribute the signal to either component. This overlap with proteinuria also accords with the baseline phenotype: patients with higher counts had more proteinuria, lower eGFR, more hypertension, and worse crude outcomes. Thus, NEU count tracks with baseline disease severity. The crescent analysis adds a pathological correlate to the clinical findings. Higher peripheral NEU counts were associated with C1 and C2 lesions relative to C0, and these associations remained detectable after adjustment for proteinuria and eGFR. This observation is compatible with the hypothesis linking NEU-related inflammatory activity to glomerular injury, for which proteinuria is a measurable marker. It provides complementary evidence from the same cohort rather than an independent validation of the hypothesized relationship.

Proteinuria and baseline eGFR are established prognostic indicators in IgAN (21–23); however, adjustment for them addresses an association conditional on the measured kidney disease. When examining factors that may act upstream of kidney injury, adjustment for a marker potentially reflecting downstream kidney injury may attenuate an association by conditioning on part of the disease process (24, 26). Chronic Renal Insufficiency Cohort (CRIC) investigators considered albuminuria as a potential intermediate between risk factors and chronic kidney disease (CKD) progression and therefore examined associations with and without albuminuria adjustment (28, 29). In studies of *APOL1*-associated CKD progression, proteinuria was omitted from the primary adjustment model because it could mediate the genotype–outcome association (30). In the Ramipril Efficacy in Nephropathy (REIN) trial, the reduction in progression risk with ramipril remained significant after adjustment for blood pressure changes but not after adjustment for changes in proteinuria (31). This rationale is relevant to our study: if proteinuria reflects glomerular injury linked to NEU-related inflammation, adjusting for it could attenuate the NEU–outcome association without excluding a biological contribution. Sex provides an illustration within our cohort because it precedes kidney disease: the adverse male associations likewise weakened or shifted to below an HR of 1 after adjustment for kidney disease markers (**Figure 3A**). The NEU associations were more sensitive to proteinuria adjustment, whereas the sex associations changed more after eGFR adjustment. Published IgAN studies also showed that sex associations depend on the comparison and adjustment conditions (32, 33). Attenuation or reversal after conditioning on kidney disease markers therefore cannot by itself be equated with loss of biological relevance or a protective effect. The sex comparison illustrates this interpretive principle without establishing that NEU lies upstream of kidney injury.

The greater sensitivity of the NEU association to proteinuria adjustment motivated the exploratory probability component analysis. For NEU ≥4.0 *versus* < 4.0 × 10^9^/L, the proteinuria-related components of the model-estimated 10-year outcome probability differences were positive for both outcomes (**Figure 3B**). When both groups were assigned the lower-NEU group’s modeled proteinuria distribution, no clear remaining difference was detected, and both remaining component CIs included zero. Sex also had positive proteinuria-related components and retained a positive remaining component for ≥50% eGFR decline. Whether an interval includes zero does not itself establish a difference between the NEU and sex components or their mechanisms. These probability components supplement the conditional associations in **Figure 3A**; they do not decompose the HR attenuation or estimate an eGFR-related probability component. The continuous-spline sensitivity analysis showed the same qualitative pattern as the binary NEU analysis. Separately, the attenuation after proteinuria adjustment persisted with additional adjustment for BMI, smoking, or CRP and when adjustment-variable coefficients were allowed to vary over follow-up. These findings support the consistency of the observed pattern across the analyses performed.

Genetic evidence implicates NEU cell functions in IgAN. IgAN-associated loci include *FCAR*, *ITGAM*/*ITGAX*, *LYN*, *CARD9*, *PADI4*, and the *DEFA* region (5–8). Studies of *DEFA* variants, copy number, and circulating α-defensins provide further links to innate immune responses (9–11, 34). These processes are relevant to NEU responses and include IgA immune complex recognition through FcαRI/CD89, β2-integrin-dependent adhesion and trafficking, inflammatory signaling, chromatin citrullination during NET formation, and antimicrobial effector activity (12, 13). FcαRI signaling can generate activating or inhibitory responses depending on context (12, 35). Studies of soluble CD89–IgA complexes have also reported associations with adult, transplant, and childhood IgAN phenotypes, although findings vary across settings (36–38). Together, these observations support the investigation of NEU as part of the broader myeloid response in IgAN. Locus-level associations do not specify the responsible effector cell or imply that higher circulating NEU counts represent genetically determined hyperreactivity.

Functional studies provide complementary evidence at several levels. NEU can release granule proteins, reactive oxygen species, and NETs that contribute to tissue injury (16). In experiments outside IgAN, IgA immune complexes induced FcαRI-dependent NET formation, with stronger induction than IgG complexes in one comparison (17–19). In patients with IgAN, circulating citrullinated histone H3, myeloperoxidase, and NEU elastase were elevated, and citrullinated histone H3 correlated with galactose-deficient IgA1. NET-related signals were also observed in kidney tissue (20). In the experimental IgAN model reported in that study, pharmacological inhibition of PAD4-dependent NET formation reduced proteinuria and mesangial injury (20). The cited LL37 preprint reported an association between serum LL37 and kidney survival, together with experimental reductions in IgA deposition, proteinuria, and endothelial permeability after disruption of NEU-derived LL37/CRAMP or LL37-containing NETs (39). These preprint findings should be interpreted with caution. These observations support a testable link between IgA-related immune stimulation, NEU activation, and NET-associated injury. Peripheral NEU count measures cell abundance rather than activation; thus, its relationship with these functional processes requires direct assessment.

The strengths of this study include a large biopsy-confirmed cohort, long follow-up, and fixed analysis sets across sequential models. Although the NEU–outcome associations were modest, the large sample size improved precision and enabled their detection. Their clinical importance requires further evaluation. Several limitations affect the interpretation of these findings. The requirement for at least 1 year of adjudicated follow-up, except for kidney failure within the first year, may have introduced selection bias by excluding 1,765 patients with shorter or unavailable adjudicated follow-up, and the direction of any resulting bias is uncertain. NEU and proteinuria were measured around biopsy and thus their biological ordering could not be established. The absence of measurements of NEU activation, NET markers, tissue infiltration, and NEU-related genotypes also limited direct assessment of the proposed mechanism. Although report-derived crescent categories were examined, a comprehensive Oxford MEST-C assessment was not performed, and the survival models did not incorporate full MEST-C adjustment. Crescent classification depended on report wording and the assumption that an unmentioned lesion was absent, which may have introduced misclassification. Report-text checks cannot replace independent slide review. Only 157 patients were classified as C2, and the cross-sectional association with NEU does not establish biological ordering. Post-biopsy treatment initiation and modification depended on evolving disease severity, which conventional baseline adjustment could not adequately address. These limitations are particularly relevant to the interpretation of attenuation and the exploratory probability components. Attenuation can reflect mediation, confounding, or other statistical structures (40). Conditioning can introduce collider bias, and Cox HRs are non-collapsible. Causal interpretation of the *g*-computation components requires the assumptions stated in “*Materials and methods*” (27), which may be violated by unmeasured glomerular injury, infection, or treatment. Neither analysis therefore establishes a total or direct causal effect, and the CIs for the probability components do not account for confounding, selection bias, or model selection uncertainty.

Clinically, the present findings identify an accessible outcome-associated marker and a hypothesis for further investigation. We did not evaluate whether NEU improves prediction beyond the established IgAN models incorporating proteinuria and eGFR (22) because discrimination, calibration, and reclassification were not assessed. The results therefore do not provide a basis for NEU-guided treatment decisions. Prospective studies should obtain repeated NEU and proteinuria measurements, record the treatment timing and dose, and evaluate whether NEU trajectories add predictive information. Mechanistic validation and evaluation of incremental predictive performance address separate questions and are both needed to define the clinical role of NEU.

## Data Availability

The raw data supporting the conclusions of this article will be made available by the authors, without undue reservation.
